# Laboratory Evaluation of a Novel Insecticide, Isocycloseram, Against the Common Bed Bug (*Cimex lectularius* L.) (Hemiptera: Cimicidae)

**DOI:** 10.3390/insects16020200

**Published:** 2025-02-12

**Authors:** Xiaodan Pan, Souvic Sarker, Changlu Wang

**Affiliations:** Department of Entomology, Rutgers-The State University of New Jersey, 96 Lipman Dr., New Brunswick, NJ 08901, USA; xiaodan.pan@rutgers.edu (X.P.); souvicsarker08@gmail.com (S.S.)

**Keywords:** insecticide spray, *Cimex lectularius*, efficacy, surface type, aging

## Abstract

Isocycloseram is a novel insecticide belonging to the class of isoxazoline (IRAC MoA Group 30) that exhibits excellent efficacy and selectivity against invertebrate pests. We evaluated the efficacy of two 0.1% isocycloseram formulations against a laboratory and four insecticide-resistant field strains of common bed bugs (*Cimex lectularius* L.) (Hemiptera: Cimicidae) by direct spray and forced exposure on three surfaces (fabric, vinyl tiles, and unpainted pinewood). Their efficacy was compared with that of five commercial insecticides including Demand CS (0.03% λ-cyhalothrin), Temprid FX (0.05% imidacloprid and 0.025% β-cyfluthrin), Crossfire (0.4% clothianidin, 0.01% metofluthrin, and 1.0% piperonyl butoxide), Bedlam Plus (0.4% d-phenothrin, 1.0% MGK 264, and 0.05% imidacloprid), and PT Phantom II (0.5% chlorfenapyr). Both isocycloseram 45 SC and 400 SC formulations were the most effective among the tested insecticides. The isocycloseram 45 SC formulation caused faster mortality than the 400 SC formulation. Four hours of exposure to 0.025, 0.05, and 0.1% isocycloseram 45 SC residue on porous and non-porous surfaces caused 100% bed bug mortality after 3–4 days. Exposure to 30-day-aged 0.1% isocycloseram 45 SC residue for 4 h still caused 100% mortality, compared to 0–73% mortality by the five commercial insecticides. Isocycloseram is a highly effective compound for controlling resistant *C. lectularius* populations.

## Simple Summary

During the last two decades, the common bed bug (*Cimex lectularius* L.) (Hemiptera: Cimicidae) resurged into a common urban pest worldwide. Insecticide sprays are commonly used to manage this pest. However, most of the available insecticide sprays registered for bed bugs are not very effective due to insecticide resistance. Bed bugs are considered one of the most difficult urban pests to control. There is strong interest in developing novel and effective materials and methods to combat the widespread bed bug infestations. Isocycloseram is a novel insecticide in the class isoxazoline that shows excellent efficacy and selectivity against invertebrate pests. We evaluated its efficacy against different strains of common bed bugs by exposing bed bugs to dry residue on different surfaces and direct spray. Both isocycloseram formulations (400 SC and 45 SC) are highly effective against bed bugs, with 45 SC causing faster mortality than 400 SC. The 30-day-aged dry residue of 45 SC did not show a significant loss of efficacy. It caused 100% mortality to resistant bed bugs and was much more effective than the five selected commercial insecticides. We conclude isocycloseram is a promising insecticide for controlling the common bed bugs.

## 1. Introduction

After around 25 years of resurgence in the U.S., the common bed bug (*Cimex lectularius* L.) (Hemiptera: Cimicidae) remains common and one of the most challenging urban pests to eliminate [1,2]. They usually hide in small and dark places, such as cracks, crevices, bed linens and bed skirts, seams of mattresses, box springs, upholstered furniture, baseboards, electrical outlets, etc. They bite during the night, causing lesions, anxiety, nightmares, phobia, hypervigilance, and insomnia [3]. The lack of effective insecticides is considered one of the major factors contributing to the bed bug resurgence and control difficulties [4,5]. Bed bugs are controlled by both chemical and non-chemical methods, such as insecticide sprays and dusts, fumigation, heat or cold treatment, and steam [6]. Among all the management tools, insecticide application is the most commonly used tool against bed bugs by pest management professionals (PMPs). More than 12 classes of insecticides have been used to control bed bugs, with pyrethroids being the most widely used [7]. Commonly used formulations are liquid sprays, pressurized aerosols, and dusts. The liquid spray formulation is further broken down into capsule suspensions (CS), emulsifiable concentrates (EC), suspension concentrates (SC), microemulsion (ME), microencapsulations (M), water-dispersible granules (WDG), and wettable powders (WP) [8].

A recent survey of 102 U.S. pest control companies found that 83% preferred liquid formulation for treating bed bugs [9]. Despite the numerous insecticides available for bed bugs, highly effective insecticides are lacking due to the prevalence of bed bug resistance to different insecticides including organophosphates [10], pyrethroids [5,11,12], neonicotinoids [13], and phenylpyrazole (fipronil) [14]. As a result, dry residues are generally not very effective against field populations of bed bugs. Residues of four products containing pyrethroid alone or a combination of pyrethroid and neonicotinoid caused ≤70% mortality on different substrates in a laboratory study [15]. A recent study demonstrated that long-lasting insecticide-treated nets (LLIN) treated with permethrin, deltamethrin, and chlorfenapyr were ineffective in eliminating *C. lectularius* [16]. Among the insecticide dusts, only silica gel dust has demonstrated high efficacy after exposure to treated surfaces [17] or treated harborages [18]. However, dust formulation is difficult to apply and can easily result in airborne particles that translocate to non-targeted areas. A study indicated that dust aerosol was effective against *C. lectularius* for at least 21 days [19]. A combination of non-chemical and chemical methods is generally required to effectively control bed bug infestations [3,7]. Still, weeks or months are often required to eliminate an infestation [20,21].

Isocycloseram, also known as PLINAZOLIN^®^ technology, belongs to a novel class of isoxazolines. It is categorized under the IRAC Mode of Action Group 30 along with meta-diamides (GABA-gated chloride channel allosteric modulators) [22]. Its residue is highly effective against arthropod pests, such as the fall armyworm (*Spodoptera frugiperda* [Smith]) (Lepidoptera: Noctuidae) [23], the Colorado potato beetle (*Leptinotarsa decemlineata* [Say]) (Coleoptera: Chrysomelidae) [24], and the western flower thrip (*Frankliniella occidentalis* [Pergande]) (Thysanoptera: Thripidae) [25]. Isocycloseram granular bait is highly effective against leaf-cutting ants (genus *Atta*) (Hymenoptera: Formicidae) both under laboratory and field conditions [26]. Isocycloseram was also tested effective as seed protection of wheat and barley against the sugarbeet wireworm (*Limonius californicus* [Mannerheim]) (Coleoptera: Elateridae) [27]. A recent study found that isocycloseram gel bait and spray were highly effective against both laboratory and field-collected strains of German cockroaches (*Blattella germanica* [L.]) (Blattodea: Ectobiidae) [28]. Another study found that isocycloseram was very effective against several urban pests, including *B. germanica*, *C. lectularius*, Argentine ants (*Linepithema humile* [Mayr]) (Hymenoptera: Formicidae), Pharaoh ants (*Monomorium pharaonis* [L.]) (Hymenoptera: Formicidae), Eastern subterranean termites (*Reticulitermes flavipes* [Kollar]) (Blattodea: Rhinotermitidae), Formosan subterranean termites (*Coptotermes formosanus* Shiraki) (Blattodea: Rhinotermitidae), and Asian malaria mosquitoes (*Anopheles stephensi* Liston) (Diptera: Culicidae) [29].

To better understand the efficacy of liquid isocycloseram formulations against the common bed bug, we conducted a series of laboratory evaluations using laboratory and insecticide-resistant field *C. lectularius* strains. We hypothesized that isocycloseram provides much higher efficacy compared to the existing registered liquid or aerosol insecticides based on previous studies against various invertebrates including urban pests such as ants, bed bugs, cockroaches, and termites. Here we report results on: (1) the residual efficacy of two isocycloseram SC formulations compared with selected commercial insecticide products against *C. lectularius*; (2) the residual efficacy of different rates of isocycloseram 45 SC (0.1, 0.05, and 0.025%) against *C. lectularius* on porous (fabric, pinewood) and non-porous (ceramic tile) surfaces; and (3) the direct spray efficacy of 0.05 and 0.025% isocycloseram 45 SC against *C. lectularius*. The results of these experiments will help determine the future application rates for treating bed bug infestations.

## 2. Materials and Methods

### 2.1. Bed Bug Strains

Five strains of *C. lectularius* were used in this study. The Ft. Dix strain (or Harlan strain in other publications) is susceptible to insecticides and has been kept in the laboratory without insecticide exposure since 1973 [30]. The four field strains and their collection years are Canfield (2018), Irvington 624-5G (2013), Masiello (2016), and New Brunswick (2021). All of them were collected from low-income apartment buildings in New Jersey, USA. All field-collected bed bug strains exhibited high resistance to deltamethrin except for the Masiello strain, which showed a low-level resistance. All of them also exhibited low-level resistance to both acetamiprid and imidacloprid. The Irvington 624-5G strain also showed high resistance to neonicotinoid–pyrethroid mixture insecticides [31]. All bed bugs were fed with defibrinated rabbit blood (Hemostat Laboratories, Dixon, CA, USA) seven days before treatment using a Hemotek membrane-feeding system (Discovery Workshops, Accrington, UK). Detailed bed bug rearing methods were described by Ranabhat and Wang [17].

Bed bug adult males were tested in this study, although adult females were included in Experiment 1. Bed bugs were placed into Petri dishes (d = 5.5 cm, h = 1.5 cm) lined with filter paper (Grade P8, Fisher Scientific, Pittsburgh, PA, USA) using soft forceps (BioQuip Products, Rancho Dominguez, CA, USA). After insecticide exposure, bed bugs were transferred back to their previous Petri dishes except those in Experiment 4, which were transferred to a new Petri dish. The Petri dishes with bed bugs were kept in an incubator at 26 °C and 12:12 D:L photoperiod before and after treatment.

### 2.2. Insecticides and Test Surfaces

The insecticides tested in this study are shown in Table 1. They include two formulations of isocycloseram and five commercial insecticides labeled for bed bug treatment. Isocycloseram 400 SC and isocycloseram 45 SC have the same active and inert ingredients but different rates. The 400 SC formulation had a much higher concentration (8.88×) of isocycloseram than the 45 SC formulation. All products were provided by Syngenta. Fabric (100% cotton Waverly inspirations Walmart stores, Inc., Bentonville, AR, USA) was washed with detergent water and dried at room temperature before cutting into 4 × 4 inch (10.16 × 10.16 cm) pieces. Then, they were attached to 4 × 4 inch (10.16 × 10.16 cm) steel panels with a glue stick to maintain a flat surface. White glazed ceramic tiles (4.25 × 4.25 inch, or 10.80 × 10.80 cm, Jeffrey Court Tile, Norco, CA, USA) were purchased from Home Depot (Milltown, NJ, USA). Unpainted pinewood (2.54 cm thick) and vinyl tiles (Armstrong Civic, Armstrong World Industries Canada Ltd., Montreal, QC, Canada) were also purchased from Home Depot (Milltown, NJ, USA). They were cut into 3.5 × 4 inch (8.89 × 10.16 cm) and 4 × 4 inch (10.16 × 10.16 cm), respectively.

### 2.3. Experimental Setup

#### 2.3.1. Experiment 1: Residual Efficacy of 0.1% Isocycloseram 400 SC Against Bed Bugs on Three Types of Surfaces

This was an initial evaluation of the efficacy of 0.1% isocycloseram 400 SC. Three types of surfaces (fabric, vinyl tiles, and unpainted pinewood) and two bed bug strains (Ft. Dix and Irvington 624-5G) were used in this experiment. The 0.1% dilution of isocycloseram 400 SC was prepared by mixing 5 mL of isocycloseram 400 SC with 1 L of tap water in one 1-gallon B&G stainless sprayer (B&G Equipment Company, Jackson, GA, USA) immediately before treatment. A second 1-gallon B&G stainless sprayer with tap water was used as control. During insecticide application, the sprayer nozzle was about 20 cm above each panel and passed each panel 3–5 times to yield approximately 1.1 gallon/1000 ft^2^ application rate. One panel was treated each time.

The treated panels were left on a shelf or in white trays in the laboratory for one day. Then, 18–23 adult bed bugs (at least 15 males) were released on each treated surface and confined within a plastic ring (d = 8.6–8.8 cm) for four hours in a walk-in chamber with only a red light on (25 W, General Electric, Fairfield, CT, USA). The interior surface of each confinement ring was coated with Insect-A-Slip (PTFE Fluoropolymer Dispersion, BioQuip Products, Rancho Dominguez, CA, USA) to prevent bed bugs from escaping. There were five replications for each surface/treatment combination. The bed bugs were kept in a walk-in chamber with a 12:12 D:L photoperiod. The temperature and the relative humidity were recorded by a HOBO External Temp/RH data logger (UX100-023A) (Onset Computer Corporation, Bourne, MA, USA); they were 25.3 ± 0.0 °C and 20.7 ± 0.1%.

Bed bugs were considered dead if they did not move when probed with forceps. Bed bugs were considered “knockdown” if their legs could move but could not crawl normally. The numbers of dead, knockdown, and live bed bugs were recorded right after exposure for 4 h and daily for seven days. This experiment was conducted from December 2020 to January 2021.

#### 2.3.2. Experiment 2: Comparison of 0.1% Isocycloseram 400 SC and 0.1% Isocycloseram 45 SC with Five Commercial Insecticides Against Bed Bugs

All insecticides were tested on fabric surfaces in this experiment. In the 1st test using Ft. Dix and Canfield strains, isocycloseram 400 SC, Demand CS, Temprid FX, and Crossfire were diluted into a one-gallon B&G sprayer with 1 L tap water following their label rates (Table 1). Bedlam Plus aerosol was sprayed directly on the panels after shaking. PT Phantom II aerosol was sprayed using an application straw that comes with the product. The sprayer nozzle was positioned 30 cm above the panels, except Bedlam Plus, which was approximately 25 cm above the panels. The nozzle passed each panel 2–4 times to yield 1.0 gallon/1000 ft^2^. The control surfaces were treated with tap water using a different B&G sprayer. A total of 20 bed bug adult males were used in each replicate. The bed bug confinement method was the same as in Experiment 1. Each treatment had four replicates. The numbers of dead, knockdown, and live bed bugs were recorded at the end of four hours of exposure and daily for seven days. The mean temperature during the test period was 24.7 ± 0.1 °C. This test was conducted in December 2021.

In the 2nd test, a different isocycloseram formulation (45 SC), application method, and bed bug strain (Masiello) were used. In addition, fresh and aged residues were tested. The isocycloseram 45 SC, Demand CS, Temprid FX, and Crossfire were diluted into 200 mL beakers and sprayed with a Potter spray tower (Burkard Scientific Ltd., Herts, UK) at the rate of 1.0 gallon/1000 ft^2^. The nozzle of Bedlam Plus and the end of the application straw attached to PT Phantom II were positioned 30 cm above each panel and passed 4 times. All treated panels were stored for 1 day (21–23 h) or 30 days before use. There were 19−22 bed bug adult males in each replicate and five replicates in each treatment. The bed bug confinement method was the same as in the previous test. The numbers of dead, knockdown, and live bed bugs were recorded at the end of four hours of exposure and daily for seven days. The mean temperature during the experiment period was 27.2 ± 0.1 °C. This test was conducted in July and August 2024.

#### 2.3.3. Experiment 3: Residual Efficacy of Two Isocycloseram Formulations Against Field Strain (Canfield) Bed Bugs on Two Types of Surfaces

Both formulations of 0.1% isocycloseram caused high bed bug mortality from previous experiments. This experiment was designed to compare the residual efficacy of 0.1% isocycloseram 400 SC, 0.1% isocycloseram 45 SC, 0.05% isocycloseram 45 SC, and 0.025% isocycloseram 45 SC against Canfield strain bed bugs on fabric and glazed ceramic tiles. All isocycloseram products were diluted into 200 mL beakers at the desired rates and sprayed with a Potter spray tower at 1.1 gallon/1000 ft^2^. Tap water was used in the control, and Demand CS was used as a positive control.

A total of 14–17 bed bug males were used in each replicate. There were five replicates in each treatment. The bed bug confinement method was the same as in previous experiments. The mean temperature during the experiment was 26.8 ± 0.1 °C. The numbers of dead, knockdown, and live bed bugs were recorded at the end of four hours of exposure and daily for seven days. This experiment was conducted in February 2022.

#### 2.3.4. Experiment 4: Efficacy of 0.05 and 0.025% Isocycloseram 45 SC Direct Spray Against Bed Bugs

The previous experiment found that all tested isocycloseram 45 SC rates caused >90% bed bug mortality at 2 d post-treatment. Thus, this experiment was designed to test the direct spray efficacy using the two lower rates (0.05 and 0.025%) of isocycloseram 45 SC. Ft. Dix and New Brunswick strains of bed bugs were tested. A total of 15 bed bug males were used in each replicate, with five replicates in each treatment. Immediately after spraying (Test 1) or four hours after spraying (Test 2), all bed bugs were transferred to a new Petri dish (d = 5.5 cm) lined with filter paper. In Test 1, the bed bugs were transferred to clean dishes less than 5 min after direct spray treatment. The numbers of dead, knockdown, and live bed bugs were recorded right after transferring into new Petri dishes and daily for seven days. This experiment was conducted in a walk-in chamber at 26.1 ± 0.1 °C, 24.1 ± 0.5% RH in November 2023.

#### 2.3.5. Experiment 5: Residual Efficacy of 0.025% Isocycloseram 45 SC Against Bed Bugs on Three Types of Surfaces

The previous two experiments showed that 0.025% and 0.05% isocycloseram 45 SC caused high bed bug mortality as dry residue or direct spray. Therefore, this experiment was designed to further test the efficacy of 0.025% isocycloseram 45 SC against two bed bug strains (Ft. Dix and New Brunswick) on three types of surfaces (fabric, vinyl tiles, and unpainted pinewood). The insecticide was applied using a Potter spray tower at a 1.0 gallon/1000 ft^2^ rate. Treated panels were kept in the laboratory for approximately 28 h before use.

A total of 20 bed bug adult males were confined to each treated panel for four hours. The bed bug confinement method was the same as in previous experiments. There were five replicates in each treatment. The numbers of dead, knockdown, and live bed bugs were recorded at the end of four hours of exposure and daily for seven days. This experiment was conducted at 25.0 ± 0.0 °C, 49.5 ± 0.1% RH in July 2023.

### 2.4. Data Analysis

The knockdown bed bugs were treated as dead in this study because none recovered. Mean mortality was calculated and corrected using Abbott’s formula [32]. Non-normally distributed data were arcsine square root transformed to meet normal distribution. Analysis of Variance (ANOVA) was used to analyze mortality data. Means were separated by Tukey’s HSD test. Nonparametric Wilcoxon Rank-Sum test was used to analyze data that were not normally distributed even after transformation. Survivorship of the two strains on each surface in Experiments 1 and 5 was calculated with the Kaplan–Meier method and compared with log-rank tests. Survivorship on each surface was also compared among treatments in Experiment 3. All analyses were performed using SAS software version 9.4 [33].

## 3. Results

### 3.1. Residual Efficacy of 0.1% Isocycloseram 400 SC Against Bed Bugs on Three Types of Surfaces

**Figure 1:**
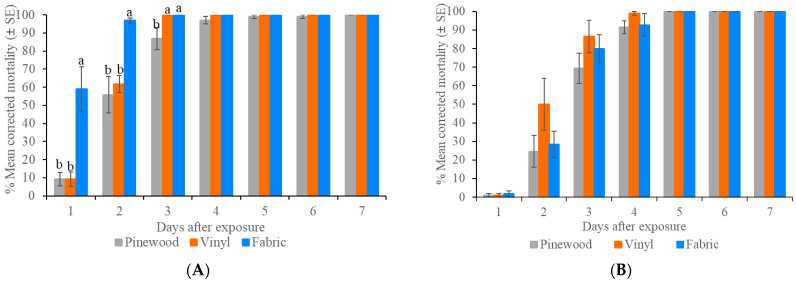
Residual efficacy of 0.1% isocycloseram 400 SC against two bed bug strains on three surfaces. (**A**) Ft. Dix strain; (**B**) Irvington 624-5G strain. During each period, different letters above the bars indicate significant differences (Tukey’s HSD test or Wilcoxon Rank-Sum test, *p* < 0.05). For Irvington 624-5G strain, the surface type had no significant effect on the mean corrected mortality (*p* > 0.05).

For the Ft. Dix strain of bed bugs, the mean corrected bed bug mortality reached 100% on unpainted pinewood, fabric, and vinyl tiles at 7, 3, and 3 d post-treatment, respectively (Figure 1). The mean mortality in the control was 1–2% on three surfaces at 3 d post-treatment. There were significant differences in the mean corrected mortality among the three surfaces at 1 d (*F* = 12.86, df = 2, *p* = 0.001), 2 d (*F* = 11.83, df = 2, *p* = 0.002), and 3 d (ꭓ^2^ = 9.88, df = 2, *p* = 0.007).

For the Irvington 624-5G strain of bed bugs, the mean corrected bed bug mortality reached 100% on all three surfaces at 5 d post-treatment (Figure 1). There were no significant differences in the mean corrected mortality among the three surfaces at any observation period (*p* > 0.05). The mean mortality in the control was 2–8% on the three surfaces at 7 d post-treatment. Compared with the Ft. Dix strain (laboratory strain), the field strain exhibited slower mortality on all three tested surfaces (log-rank test, *p* < 0.05).

### 3.2. Comparison of 0.1% Isocycloseram 400 SC and 0.1% Isocycloseram 45 SC with Five Commercial Insecticides Against Bed Bugs

**Figure 2:**
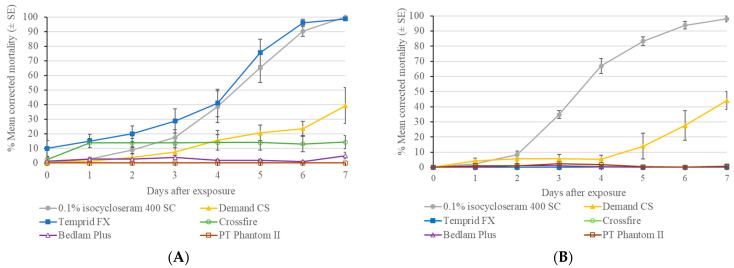
Residual efficacy of six insecticides against two strains of bed bugs on fabric surface. (**A**) Ft. Dix strain; (**B**) Canfield strain.

In Test 1 evaluating the comparative efficacy of 0.1% isocycloseram 400 SC with commercial insecticides, only 0.1% isocycloseram 400 SC and Temprid FX caused high mortality to Ft. Dix strain bed bugs at 7 d post-treatment (Figure 2). They caused significantly higher bed bug mortality than the other insecticides (χ^2^ = 22.33, df = 5, *p* < 0.001). Other treatments caused 0–39% mean mortality. Demand CS caused significantly higher bed bug mortality than Crossfire. Neither Bedlam Plus nor PT Phantom II caused significant mortality to bed bugs. After 1 d to 3 d exposure, Temprid FX caused significantly higher mortality than 0.1% isocycloseram 400 SC (*p* < 0.05). However, no difference was observed between them from 4 d (*p* > 0.05). The mean mortality in the control for the Ft. Dix strain was 4 ± 2% at 7 d post-treatment.

For the Canfield strain, the 0.1% isocycloseram 400 SC was the only insecticide causing high (98 ± 2%) mortality at 7 d post-treatment (Figure 2). Demand CS only caused 44 ± 6% bed bug mortality at 7 d, which was significantly lower than that in the 0.1% isocycloseram 400 SC treatment (χ^2^ = 21.52, df = 5, *p* < 0.001). Temprid FX, Crossfire, Bedlam Plus, and PT Phantom II were ineffective in killing the Canfield strain of bed bugs. The mortality in the control was 13 ± 1% at 7 d post-treatment.

**Figure 3:**
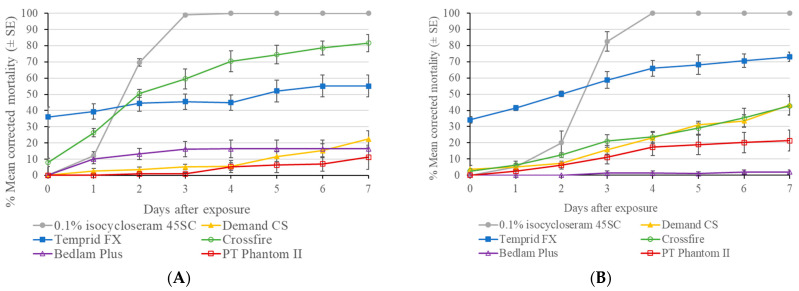
Residual efficacy 0.1% isocycloseram 45 SC and five commercial insecticides against a field strain (Masiello) bed bugs on fabric surface. (**A**) Fresh residue; (**B**) 30-day-aged residue.

In Test 2 evaluating the comparative efficacy of 0.1% isocycloseram 45 SC with commercial insecticides, the 7 d post-treatment mean corrected mortality ranged from 11% to 100%. They were significantly different (χ^2^ = 25.04, df = 5, *p* < 0.001) (Figure 3). The 0.1% isocycloseram 45 SC was the only insecticide causing 100% bed bug mortality, which happened at 4 d post-treatment. Crossfire was also very effective against the Masiello strain of bed bugs. However, it required 7 days to kill 80% of the bed bugs. Temprid FX was the third most effective insecticide. Demand CS, Bedlam Plus, and PT Phantom II caused 11–22% bed bug mortality at 7 d, and they were not significantly different (χ^2^ = 2.46, df = 2, *p* = 0.29). The mean bed bug mortality in the control was 2 ± 1% at 7 d post-treatment.

In Test 2 with 30-day-aged insecticide residues, the 7 d post-treatment corrected mortality ranged from 21% to 100%, and they were significantly different (*F* = 59.24, df = 5, *p* < 0.001) (Figure 3). The 0.1% isocycloseram 45 SC was the only insecticide causing 100% bed bug mortality, which happened at 4 d post-treatment. The mortality in the control was 1 ± 1% at 7 d post-treatment. Compared with the fresh residues, isocycloseram 45 SC and Temprid FX did not exhibit a significant loss in their efficacy. However, 30-day-aged Crossfire residue caused much lower mortality than the fresh residue (43 ± 6% vs. 82 ± 5%).

### 3.3. Residual Efficacy of Two Isocycloseram Formulations Against a Field Strain Bed Bugs on Two Types of Surfaces

**Figure 4:**
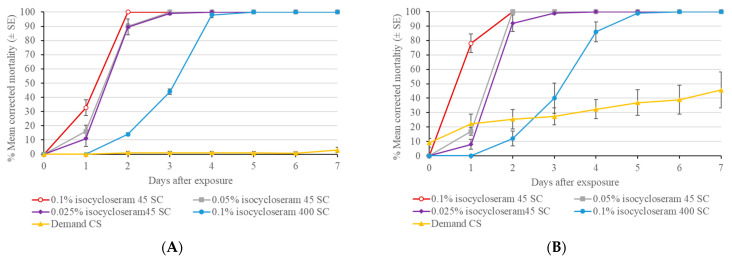
Residual efficacy of two isocycloseram formulations against a field strain (Canfield) bed bugs on two types of surfaces. (**A**) Fabric surface; (**B**) glazed ceramic surface.

On porous fabric surface, the 0.1% isocycloseram 400 SC and the three rates of isocycloseram 45 SC (0.1, 0.05, and 0.025%) were highly effective against bed bugs, causing 100% mortality after 2–5 d (Figure 4). In comparison, Demand CS only caused 3 ± 2% bed bug mortality at 7 d. The three rates of 45 SC formulation caused faster mortality than 0.1% isocycloseram 400 SC, and 0.1% 45 SC caused faster mortality than 0.025% 45 SC treatment (log-rank test, *p* < 0.05). The mean mortality in the control was 2 ± 1% at 7 d post-treatment.

On non-porous ceramic tile surface, the 0.1% isocycloseram 400 SC and the three rates of isocycloseram 45 SC (0.1, 0.05, and 0.025%) were highly effective in killing bed bugs, causing 100% mortality at 6, 2, 2, and 4 d, respectively (Figure 4). Demand CS only caused 46 ± 13% mortality at 7 d, which was significantly lower than that in all isocycloseram treatments from 2 d (Wilcoxon Rank-Sum test, *p* < 0.05). Similar to the results on the fabric surface, the three rates of 45 SC formulation caused faster mortality than 0.1% isocycloseram 400 SC. In addition, 0.1% 45 SC caused faster mortality than 0.05% and 0.025% 45 SC treatment (log-rank test, *p* < 0.05). The mean mortality in the control was 2 ± 1% at 7 d post-treatment.

### 3.4. Efficacy of 0.05 and 0.025% Isocycloseram 45 SC Direct Spray Against Bed Bugs

**Figure 5:**
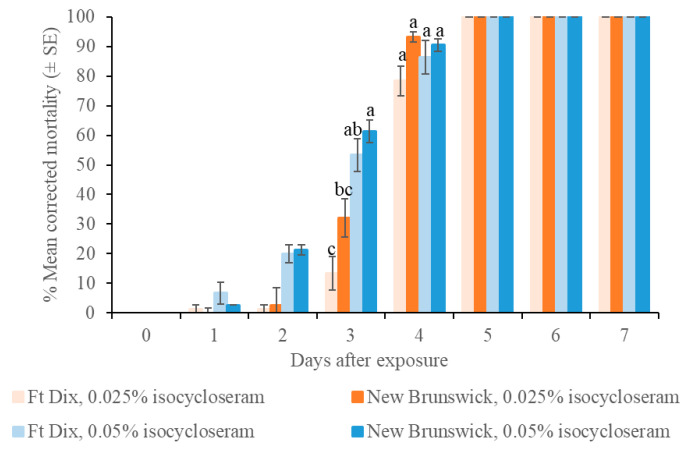
Efficacy of isocycloseram 45 SC direct spray against two strains of bed bugs. At each period, different letters above the bars indicate significant differences (Tukey’s test, *p* < 0.05).

In Test 1, in which bed bugs were immediately transferred into new Petri dishes after direct spray, both the 0.05 and 0.025% rates of isocycloseram 45 SC caused 100% mortality in both the Ft. Dix and New Brunswick bed bug strains at 5 d post-treatment (Figure 5). The higher rate of isocycloseram 45 SC (0.05%) caused significantly higher bed bug mortality than the lower rate (0.025%) at 2 d and 3 d. In Test 2 where bed bugs were transferred into new Petri dishes 4 h after direct spray, both 0.05 and 0.025% rates of isocycloseram 45 SC caused 100% mortality in both the Ft. Dix and New Brunswick bed bug strains at 5 d post-treatment, the results were similar to those of Test 1. In both tests, the control mortality of the Ft. Dix and New Brunswick strains of bed bugs was 1–3% at 7 d post-treatment.

### 3.5. Residual Efficacy of 0.025% Isocycloseram 45 SC Against Bed Bugs on Three Types of Surfaces

**Figure 6:**
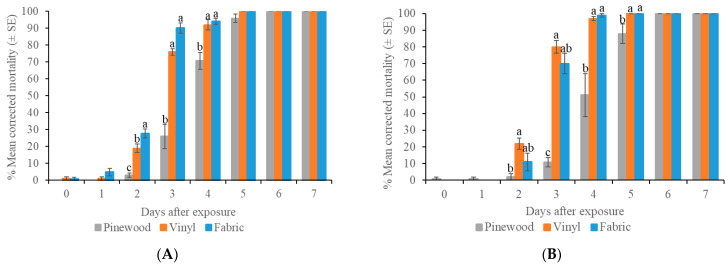
Time mortality response of strains of bed bugs after 4 h exposure to 0.025% isocycloseram 45 SC-treated surfaces. (**A**) Ft. Dix strain; (**B**) New Brunswick strain. Different letters indicate significant differences among surfaces at the same day (Tukey’s test/Wilcoxon test, *p* < 0.05).

For Ft. Dix strain bed bugs, the mean corrected bed bug mortality reached 100% on fabric, vinyl tiles, and unpainted pinewood after 5 or 6 days (Figure 6). There were significant differences in the mean corrected bed bug mortality among the three surfaces at 2 d (*F* = 33.07, df = 2, *p* < 0.001), 3 d (*F* = 51.27, df = 2, *p* < 0.007), and 4 d (*F* = 9.11, df = 2, *p* = 0.004). The mean mortality in the control was 1–9% at 7 d post-treatment.

For New Brunswick strain bed bugs, the mean corrected bed bug mortality reached 100% on fabric, vinyl tiles, and unpainted pinewood after 5 or 6 days (Figure 6). The mean corrected bed bug mortality on vinyl tiles was significantly higher than that on pinewood during 2–5 d (Tukey’s test/Wilcoxon test, *p* < 0.05). In addition, mortality on the fabric surface was significantly higher than that on pinewood at 4 and 5 d. The control mortality was 5–8% at 7 d post-treatment.

There were no significant differences in the mean corrected mortality between the Ft. Dix and New Brunswick strains on each surface (*p* > 0.05). Thus, both strains were susceptible to 0.025% isocycloseram 45 SC. Compared with the Ft. Dix strain (laboratory strain), the field strain exhibited slower mortality on fabric and pinewood surfaces (log-rank test, *p* < 0.05).

## 4. Discussion

This study confirmed that isocycloseram is a highly effective new insecticide against *C. lectularius*. The 45 SC formulation is more effective than the 400 SC formulation. When compared with five other commercial insecticides, the 0.1% isocycloseram (either 45 SC or 400 SC formulation) was the only one causing >90% mortality to two resistant bed bug strains (Canfield and Masiello). The aging experiment shows that 0.1% isocycloseram 45 SC was the only tested insecticide causing 100% mortality after 30-day aging. Fresh dry residues of 0.05 and 0.025% isocycloseram 45 SC formulation caused 100% bed bug mortality within five days on both porous (fabric) and nonporous (ceramic) surfaces. A recent study on 0.2% isocycloseram 400 SC found high efficacy (>95% mortality) against both laboratory-strain and field-strain *C. lectularius* on three surfaces (fabric, linoleum tile, and unpainted pinewood) [29]. Compared with that study, we used a much lower rate (0.1%) of isocycloseram 400 SC. Similar studies on commercial liquid insecticides against bed bugs did not show high efficacy. For instance, Bedlam (0.4% d-phenothrin, 1.6% MGK 264), Demand CS, and Temprid SC (0.05% imidacloprid and 0.025% beta-cyfluthrin) resulted in ≤70% mortality against *C. lectularius* on four types of substrates using the same treatment methods [15]. In another study, Temprid and Tandem (0.1% thiamethoxam and 0.03% lambda-cyhalothrin) residue on filter paper caused 0–37% mortality to field populations of tropical bed bugs (*C. hemipterus* [F.]) after 5 days of continuous exposure [34]. Our results are consistent with findings on its high insecticidal activity against another common indoor pest, the German cockroach [28].

Shikano et al. [35] found that exposure to Crossfire and Bedlam Plus residue on fabric for 15 min caused 33 and 23% mortality to a field bed bug population, respectively. We expected higher mortality against the laboratory strain. However, in the current study, Crossfire and Bedlam Plus caused ≤ 16% mortality to the Ft. Dix strain after 4 h exposure. The lack of efficacy of the dry residue of these insecticides indicates that these insecticides had little to no residual effect when applied to absorbent surfaces. Substrate type and exposure time affect the residual activity of insecticide spray and aerosol products against bed bugs [15]. Continuous exposure during the whole study period may cause higher mortality, but bed bugs rarely rest continuously on a treated surface under field conditions.

The differential performance of Temprid FX against the laboratory and two field strains (Canfield and Masiello) of bed bugs supports our previous observations of their different levels of resistance to deltamethrin [31]. In contrast, isocycloseram caused similar mortality against a laboratory strain (Ft. Dix) and two field strains (Irvington 624-5G and Canfield) with high resistance to deltamethrin (Figure 1 and Figure 2); however, the speed of mortality differed significantly between the laboratory strain and the field strain (Figure 1 and Figure 6), suggesting the lower sensitivity to isoxazoline-class insecticides in the field bed bug populations compared to the laboratory strain. Similarly, Lee et al. [28] found that a slower mortality of field German cockroach populations from isocycloseram bait treatment was observed in laboratory assays. They also found that two of the five field German cockroach populations exhibited significantly lower sensitivity to isocycloseram (resistance ratio 1.6 and 3.0×) using topical assay despite no prior history of isocycloseram exposure [28]. However, complete mortality was achieved in all tested field strains. In a recent study, it was demonstrated that even in three laboratory-susceptible populations of the German cockroach, the resistance ratios were 1.69 and 2.29, suggesting that variations in LD_50_ values can also occur among laboratory strains [29]. Findings from our study and that of Lee et al. [28] suggest that while cross-resistance between isocycloseram and other classes of insecticide is not detected, some individuals in the field bed bug and German cockroach populations are less sensitive to isocycloseram. Hakerhorn et al. [36] found insecticide exposure induced the expression of a multitude of genes in *C. lectularius*, most of which were involved in detoxification. These genetic differences could explain the delayed mortality from isocycloseram treatment in the field populations compared with that in the laboratory strain. Rotating among different classes of insecticides and incorporating non-chemical methods is recommended to delay the development of insecticide resistance [37].

Due to the hiding behavior of bed bugs, it is difficult to find bed bugs and apply insecticides directly to bed bugs during treatment. Therefore, applying a highly effective non-repellent residual insecticide is desirable to eliminate bed bug infestations efficiently and economically. Past studies have indicated that permethrin-impregnated mattress liners had very little effect against 24 strains of *C. lectularius* and *C. hemipterus* from different places in the world [12]. Another study found that permethrin-, deltamethrin-, or chlorfenapyr-treated LLIN were ineffective in controlling either susceptible or resistant strains of *C. lectularius* even with continuous exposure [16]. A combination of non-chemical methods (steam, mattress encasement, and laundering) and residual spray with pyrethroid–neonicotinoid insecticide mixtures found high reduction (89–98%) in *C. lectularius* counts in apartments, but low elimination rates after 8 weeks [20]. Isocycloseram was found to be non-repellent to German cockroaches and termites [28,29]. Its high residual efficacy combined with non-repellency and long residual life (30-day aging without significant loss in efficacy) will make it a very promising material when used as a residual spray or impregnated barrier for treating bed bugs.

Even though various non-chemical methods such as applying steam [38,39], heat [40], freezing [41], frequent laundering [42], and mattress encasement [43] are known to be effective methods, insecticide treatment is likely to continue to be the main method for controlling bed bugs by professionals in the future, due to the low cost and ease of application of pesticides (compared to non-chemical methods). The development of novel insecticides and/or advanced formulations is essential. The high efficacy of isocycloseram shown in this study indicates it may significantly improve future bed bug management if it becomes commercially available. Additional laboratory studies examining efficacy following short-term exposure and field studies are warranted to prove its effectiveness in controlling natural *Cimex* spp. (including *C. hemipterus*) infestations.

## Figures and Tables

**Table 1:** Insecticides evaluated in this study.

Product Name	Formulation	Active Ingredient(s)	Manufacturer
Isocycloseram 400 SC	SC	0.1% isocycloseram	Syngenta Crop Protection LLC, Greensboro, NC, USA
Isocycloseram 45 SC	SC	0.1, 0.05, 0.025% isocycloseram	Syngenta Crop Protection LLC, Greensboro, NC, USA
Demand CS	CS	0.03% λ-cyhalothrin	Syngenta Crop Protection LLC, Greensboro, NC, USA
Temprid FX	SC	0.05% imidacloprid, 0.025% β-cyfluthrin	Bayer Crop Science LP, Research Triangle Park, NC, USA
Crossfire	SC	0.4% clothianidin, 0.01% metofluthrin, 1.0% piperonyl butoxide	MGK Company, Minneapolis, MN, USA
Bedlam Plus	A	0.4% d-phenothrin, 1.0% MGK 264, 0.05% imidacloprid	MGK Company, Minneapolis, MN, USA
PT Phantom II	A	0.5% chlorfenapyr	BASF Corporation, Florham Park, NJ, USA

## Data Availability

Data are available upon request.
